# Robust Data Driven Model Order Estimation for Independent Component Analysis of fMRI Data with Low Contrast to Noise

**DOI:** 10.1371/journal.pone.0094943

**Published:** 2014-04-30

**Authors:** Waqas Majeed, Malcolm J. Avison

**Affiliations:** 1 Department of Electrical Engineering, Lahore University of Management Sciences, Lahore, Pakistan; 2 Vanderbilt University Institute of Imaging Science, Vanderbilt University Medical Center, Nashville, Tennessee, United States of America; 3 Department of Radiology & Radiological Sciences, Vanderbilt University Medical Center, Nashville, Tennessee, United States of America; 4 Department of Neurology, Vanderbilt University Medical Center, Nashville, Tennessee, United States of America; 5 Department of Pharmacology, Vanderbilt University Medical Center, Nashville, Tennessee, United States of America; Universiteit Gent, Belgium

## Abstract

Independent component analysis (ICA) has been successfully utilized for analysis of functional MRI (fMRI) data for task related as well as resting state studies. Although it holds the promise of becoming an unbiased data-driven analysis technique, a few choices have to be made prior to performing ICA, selection of a method for determining the number of independent components (nIC) being one of them. Choice of nIC has been shown to influence the ICA maps, and various approaches (mostly relying on information theoretic criteria) have been proposed and implemented in commonly used ICA analysis packages, such as MELODIC and GIFT. However, there has been no consensus on the optimal method for nIC selection, and many studies utilize arbitrarily chosen values for nIC. Accurate and reliable determination of true nIC is especially important in the setting where the signals of interest contribute only a small fraction of the total variance, i.e. very low contrast-to-noise ratio (CNR), and/or very focal response. In this study, we evaluate the performance of different model order selection criteria and demonstrate that the model order selected based upon bootstrap stability of principal components yields more reliable and accurate estimates of model order. We then demonstrate the utility of this fully data-driven approach to detect weak and focal stimulus-driven responses in real data. Finally, we compare the performance of different multi-run ICA approaches using pseudo-real data.

## Introduction

The inherent complexity of functional neuroimaging data has led to an increased interest in analysis techniques capable of revealing the intrinsic architecture of the data. Several factors contribute to the spatiotemporal variation found in functional neuroimaging data, such as different modes of brain activity, non-neural physiological sources such as respiratory and cardiac signals, imaging artifacts and random noise. The ability to partition the spatiotemporal variation according to the underlying sources would be the ideal way of isolating the neural activity (or any other components of interest) and developing an in-depth understanding of the functional architecture. It turns out that it is possible, at least in theory, to decompose the data into these underlying sources using Independent Component Analysis (ICA) provided the following conditions are met: 1) The underlying signal sources are statistically independent (i.e. unrelated); 2) The sources have non-Gaussian distributions; 3) The sources are mixed in a linear fashion to yield the data. An implicit assumption of ICA is that the number of observations is equal to the number of underlying sources (or alternatively, the true number of sources is known, or can be estimated). This is an important assumption that strongly influences the ICA result, and will be discussed later in the text.

The potential of ICA to blindly separate neural activity related contributions from the data in the presence of neurally irrelevant contributions has made it an attractive choice for analysis of electrophysiology and neuroimaging data e.g. electroencephalography (EEG), magnetoencephalography (MEG) and functional MRI (fMRI) [1]–[3]. For task-related fMRI experiments, ICA not only separates consistently task-related components, but can also identify the regions in the brain that are transiently activated due to the stimulus [4]. Additionally, it can be used for noise removal, thus resulting in greater functional contrast sensitivity [5]. ICA has been an attractive choice for analysis of resting state fMRI data because it does not require pre-selection of a seed time-course, thus removing the bias from the analysis. Several studies have been performed in humans and animal models that utilize ICA to identify functionally relevant networks in the brain [2], [6], [7].

In the context of functional neuroimaging data, ICA can be used in spatial or temporal domains (referred to as spatial/temporal ICA). Spatial ICA (sICA) seeks to decompose the set of images acquired at different times into a set of statistically independent images, whereas temporal ICA (tICA) decomposes the time-courses into a set of statistically independent time-courses. tICA has been used for the analysis of EEG/MEG data due to the large temporal but small spatial dimensions of the data [1]. Conversely, sICA has been the preferred choice for the analysis of fMRI data due to large spatial but small temporal dimensions. McKeown et al argued that the assumption of statistical independence of the spatial images corresponding to different sources is reasonable for fMRI, and sICA has been used to analyze task-related as well as resting state MRI data [3], [4], [7], [8]. This study focuses on sICA and we will use ICA and sICA interchangeably for the remainder of the document.

As mentioned earlier, ICA decomposes the data into as many sources (spatial maps, in the case of sICA) as the number of observations (number of images in the series). In practice, the number of images in the series (more than 100) is much larger than the plausible number of sources. Therefore, the data has to be reduced to a smaller number of images prior to ICA analysis. The most widely used way to do this is principal component analysis (PCA). PCA (in the spatial domain) decomposes the data into a set of orthogonal images, ranked according to their contribution to the total variance in the data. A reduced dataset comprising the first nIC principal components is subjected to ICA to obtain nIC independent components (ICs). nIC will be referred to as the model order henceforth. Ideally, nIC should be equal to the true number of sources in the data. However, in practice, it has to be provided as an input for the analysis. Deviations from true or “optimal” model order have been reported to affect the results. Too large values of nIC result in overfitting and spatially sparse components, whereas too small values result in spatially non-specific (global) independent components [9]. A realistic estimate of nIC is crucial, therefore, if ICA is to identify the true underlying sources. Several approaches have been used for model order selection. Information theoretic approaches such as Minimum description-length criterion (MDL), Akaike's information criterion (AIC) and Bayesian information criterion (BIC) have been implemented in popular ICA software packages and are widely used. Some less-commonly used methods (e.g. a method based upon bootstrap stability of the principal components) have also been used for ICA of fMRI data [10]. In some studies, more subjective approaches (such as arbitrary selection of nIC, and selection based on the variance explained by the first few principal components) are used [11], [12]. However, none of these approaches is globally accepted as a standard for ICA, and different studies utilize different techniques to estimate/select nIC. This raises questions about the truly data-driven and “blind” nature of ICA and the comparability of the ICA results across studies, and establishes a need for evaluation of different approaches for model order estimation.

Accurate model order (nIC) selection is the central issue in accurate data driven source mapping with ICA, and becomes increasingly critical when analyzing data sources of interest that contribute a relatively small fraction of the total variance. Such situations can arise when the CNR is small, or when the activity of interest has a very focal spatial (or very sparse temporal) distribution. Overestimation of the model order results in overfitting, whereas underestimation of the model order may result in exclusion of the contribution of the activity of interest in situations where it contributes a relatively small amount of variance to the data. In principle, the CNR of sources of interest may be increased in the data by appropriate temporal filtering [7]. However, to our knowledge, the effects of such preprocessing steps on the validity of the nIC estimated by different methods have not been studied.

In this study, we evaluated the performance of different data-driven nIC estimation approaches for simulated data, demonstrating that the model order estimated using bootstrap stability analysis (BSA) of principal components [13] provided a better estimate of the true number of components over a wide range of CNRs and preprocessing conditions. The BSA approach was used to analyze high-resolution, low CNR fMRI data from squirrel monkeys, and focal patterns of brain activity associated with stimulation of individual digits were successfully detected using two group ICA approaches. We further investigated the performance of different multi-run ICA methods on pseudo-real data. This work highlights the shortcomings of commonly used nIC estimation and multi-run/group analysis methods for ICA, and provides alternatives that result in more stable and accurate results. Further, this study suggests that ICA can be used to detect weak and focal neuronal changes in fMRI in a completely data-driven fashion with higher sensitivity and specificity (compared with hypothesis-driven activation mapping), and therefore extends the spectrum of fMRI applications for which ICA can be used.

## Theory

### 1. ICA Model and Estimation

Consider a *p* x *n* data matrix *X*, where *p* corresponds to the number of variables (time-points in the case of sICA) and n corresponds to number of observations (number of voxels for sICA – rows of *X* are obtained by concatenating voxel intensity values at different locations in the brain). The ICA model assumes that the observed variables are generated by linear combination of hidden non-Gaussian “source” variables that are statistically independent. In the context of sICA, this means that the observed images constitute a linear combination of the spatial maps of the underlying source images, i.e.
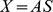



Rows of *S* correspond to the spatial maps representing different sources, and the temporal dynamics associated with the spatial maps are contained in the respective columns of *A* (the mixing matrix). ICA aims at estimating an unmixing matrix W, such that
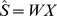



Different methods have been proposed to estimate the unmixing matrix *W*, under the assumption that the source variables have non-Gaussian distributions, such as FastICA and infomax [14]. FastICA is an iterative algorithm that estimates the rows of *W* one by one by maximizing the non-Gaussianality of rows of 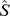. Infomax aims at estimating *W* such that the rows of 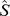 are maximally independent. Both the approaches have been shown to be theoretically equivalent [14].

### 2. Model order estimation

ICA estimates as many independent components as the number of rows in the data matrix *X*. In practice, the number of rows of *X* (number of time-points) is much greater than the plausible number of underlying sources. However, *X* is still a full rank matrix in most cases because of the white noise. Estimation of the independent components directly from matrix *X* risks over-fitting noise sources and yields spatially sparse independent components. For this reason, it is necessary to reduce the dimensionality of the data. Dimensionality reduction is typically achieved using PCA.

PCA (in the spatial domain) decomposes the data into a set of orthogonal images, ranked according to their contribution to the total variance in the data. First, the sample correlation of the data matrix is obtained: 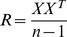. *R* is symmetric and positive semidefinite, and therefore has real, non-negative eigenvalues 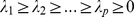 and corresponding orthogonal eigenvectors 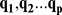. *R* can be factorized as following: 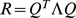, where 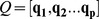 and 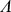 is a diagonal matrix with diagonal elements equal to 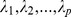. The rows of matrix 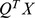
contain the principal components of data, and the fraction of the total variance of the data explained by the *i^th^* principal component is equal to 
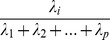
. The principal components are linear combinations of rows of *X*, and are mutually orthogonal. Therefore PCA can be seen as a whitening transform.

In the absence of noise, the number of non-zero eigenvalues would be equal to the number of independent sources. Therefore, ICA can be performed on the principal components corresponding to non-zero eigenvalues only. However, in the presence of noise, 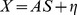, where 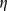 is a *p* x *n* matrix and represents the Gaussian noise contribution. In this case, the rank of *X* is greater than the true number of sources, and dimensionality reduction (by discarding some of the principal components with non-zero eigenvalues) is required to avoid overfitting. A reduced dataset comprising the first nIC principal components is then subjected to ICA to obtain nIC independent components.

Despite its potential to strongly influence the final results of ICA, there remains no consensus on the appropriate method for nIC estimation. Several approaches have been proposed and are available in ICA packages such as GIFT (http://icatb.sourceforge.net) and MELODIC (http://fsl.fmrib.ox.ac.uk/fsl/fslwiki/), and have gained popularity during the past decade [15]. These include Akaike's information criterion (AIC), minimum description-length criterion (MDL), Bayesian information criterion (BIC) and Laplace approximation to the Bayesian evidence of the model order (LAP). AIC, MDL and BIC criteria are mathematically similar and involve minimization of a cost function of the form 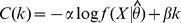, where 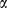 and 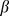 are positive constants for a given dataset, 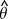 is a vector containing model parameters (eigenvalues, eigenvectors and estimated noise variance, in our case) [16], [17]. The first term rewards log-likelihood of the fitted model with *k* free parameters, whereas the second term penalizes complexity of the model (i.e. the number of free model parameters). In the context of PCA-based representation of the data, 
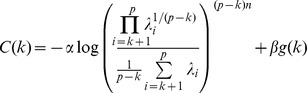
, where *k* is the number of retained principal components, and 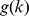
is an increasing function of *k* whose exact formula depends upon whether the data are real or complex [16], [18]. 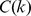 is computed for different values of *k*, and the value of *k* minimizing the cost function is chosen as nIC. In this formulation, it is assumed that 1) the additive noise is white, 2) the sources are Gaussian and 3) the observations (voxel values in an image, in our case) are independent and identically distributed [16]. The LAP criterion for model order estimation is based upon probabilistic PCA. The model order that maximizes the Laplace approximation to the model evidence is chosen as nIC [19]. LAP assumes a Gaussian distribution of the sources [19].

The model order criteria described above involve assumptions that are violated for real fMRI data. For example, voxel values within an image cannot be treated as independent variables due to the intrinsic point spread function of fMRI as well as blurring applied as a preprocessing step [18]. Additionally, the sources to be estimated are inherently non-Gaussian (which is the main assumption behind source estimation using ICA). Also, temporal filtering, when performed as a preprocessing step, can change the temporal structure of otherwise uncorrelated noise, further violating the underlying assumptions. Therefore, there is a need to explore methods that involve fewer and less restrictive assumptions about the data.

### 3. Multi-run/Group-ICA approaches

Different approaches can be used in situations where multiple datasets (collected from different subjects or different runs/sessions from the same subject) need to be collectively analyzed using ICA. The two approaches compared in this article are summarized below:

#### 3.1. Concatenation ICA with 2-step PCA reduction (ICA_cat_)

This strategy has been previously described in [3] and is one of the most widely used group ICA approaches. Data dimensionality is reduced using two-stage PCA reduction prior to ICA. First, the data from each dataset (individual runs, in our case) is reduced to *V* dimensions by selecting the principal components that correspond to the *V* largest eigenvalues. The reduced datasets are then concatenated and passed through a second stage of PCA reduction to nIC dimensions. Typically, *V* and nIC are chosen by performing model order estimation on the individual datasets and concatenated reduced dataset. ICA is performed on the reduced data and the components are then back reconstructed to obtain the components specific to the individual datasets.

#### 3.2. ICA on averaged runs (ICA_avg_)

All the preprocessed runs/subjects within a session are averaged together, and the model order is estimated for the averaged dataset. ICA is performed on the averaged dataset [20]. This method results in loss of subject/run specific information, but may result in greater effective CNR if the activity of interest is time-locked across runs/subjects (e.g. block-design experiments).

## Study Design and Methods

### 1. Study Design

First we compared performance of different nIC estimation strategies using simulated data, where the ground truth is known. Simulated data were generated with different CNRs and the following nIC estimation strategies were compared: Boostrap stability analysis of principal components (BSA), Akaike's information criterion (AIC), minimum description-length criterian (MDL), Bayesian information criterion (BIC) and Laplace approximation to the Bayesian evidence of the model order (LAP). AIC, MDL, BIC and LAP methods are implemented in widely available ICA toolboxes.

Second, we validated our choice of nIC selection strategy using low-CNR fMRI data with a known driving stimulus. The data were acquired from monkeys with tactile stimulation of digits 1 and 3 (D1 and D3), as described in previous reports [18], [21].

Third, we compared different multi-run ICA strategies using pseudo-real data.

Pseudo-real data were generated by superimposing activation on real resting state data. The purpose of utilizing pseudo-real data was to assess the ability of our methods to detect the known activation patterns with small magnitude in the presence of the structured and unstructured noise present in real datasets.

### 2. Generation of Simulated Data

Simulated datasets were generated with 15 underlying non-Gaussian sources and 300 time points. These datasets were used for optimization of nIC estimation criteria. The sources were generated using the square of a Gaussian distribution with zero mean and unit variance, while keeping the original signs [20]. The variances of the individual sources were scaled so that the variance of the i^th^ source was equal to i^2^, thus simulating the natural variation in the degree to which different sources contribute towards the total variance. The time-courses for the sources were generated from a zero-mean Gaussian distribution with unit variance, followed by low-pass filtering (0–0.1 Hz). The sources were mixed in accordance with the associated time-courses, followed by addition of white Gaussian noise with different variances to obtain different levels of percentage contribution due to the underlying sources. 50 simulated datasets were obtained for each level of noise.

Datasets generated using this method are referred to as D_sim_ datasets henceforth.

### 3. Acquisition of fMRI data

#### 3.1. Animal Preparation

All procedures were in compliance with and approved by the Institutional Animal Care and Use Committee of Vanderbilt University. Two squirrel monkeys were sedated with ketamine hydrochloride (20 mg/kg) with atropine (0.08 mg/kg) and, after intubation, ventilated with isoflurane (0.5–1.1%) delivered in a 30∶70 O_2_:NO_2_ mixture to maintain a light level of anesthesia. Animals were then placed in a custom-designed MR cradle and their heads secured with ear and head bars. Intravenous fluids (lactated ringers with 2.5% of dextrose) were infused (3 ml/kg/hr) throughout an imaging session to prevent dehydration. For cerebral blood volume (CBV) weighted contrast, a bolus 12–16 mg Fe/kg dextran coated MION contrast agent with an average particle diameter of 30 nm in saline, was injected intravenously along with 0.9% saline solution. This agent has a half-life time >6 h in blood (Zhao et al., 2003), where a steady-state condition was reached within a few minutes after injection. SpO_2_ and heart rate (Nonin, Plymouth, MN), ECG, ET-CO_2_ (22–26 mm Hg; Surgivet, Waukesha, WI), and respiratory pattern (SA instruments, Stony Brook, NY) were monitored. Rectal temperature (SA instruments) was maintained (37.5–38.5°C) via a circulating water blanket (Gaymar Industries, Orchard Park, NY). Real time monitoring of the animal was maintained from the time of induction of anesthesia until full recovery.

#### 3.2. Stimulus Presentation

Fingers were secured by gluing small pegs to the fingernails and fixing these pegs firmly in plasticine, leaving the glabrous surfaces available for vibrotactile stimulation. Piezoceramic actuators (Noliac, Kvistgaard, Denmark) delivered a vertical indentation of a 2 mm diameter probe with 0.34 mm displacement to individual distal fingerpads. The piezoactuators were driven by Grass stimulators (Grass-Telefactor, West Warwick, RI) at a rate of 8 Hz with 30 ms pulse duration. Seven alternating 30 s blocks of baseline and vibrotactile stimulation were delivered per imaging run. The MR scanner controlled the timing of the stimulus blocks consisting of simultaneous stimulation of digits 1 and 3 (D1 and D3).

#### 3.3. MRI Methods

MR imaging was performed on 9.4 T 21 cm narrow-bore Varian Inova magnet (Varian Medical Systems, Palo Alto, CA) using a 3 cm surface transmit-receive coil positioned over the somatosensory cortex. T2*-weighted gradient echo structural images (TR/TE 200/16 ms, 16 slices, 512×512 matrix; 78×78×500 µm^3^ resolution) were acquired to identify cortical venous structures that were used to locate SI cortex and provide structural features for coregistration of fMRI maps across imaging sessions. Isotropic 3D image (500×500×500 µm^3^) was collected using 3D gradient echo pulse sequence for registration across imaging sessions. For CBV-weighted fMRI, 2-shot, multi-slice gradient-echo EPI (TR/TE 750/10 ms) images were acquired beginning 10 minutes following a slow i.v. bolus of MION (12–16 mg/kg), with an increased in-plane resolution of 273×273 µm^2^ compared to our previous studies (625×625 µm^2^, [18], [22]). Triple references were used for phase correction. B0 map (τ 2 ms) at the same resolution was collected for distortion correction.

Each monkey was imaged on two different days, thus resulting in two imaging sessions per monkey. 3–6 CBV weighted runs were acquired for each session. 6 runs of resting state data were acquired from one of the monkeys (used later for generation of pseudo-real datasets).

Datasets acquired using this method are referred to as D_real_ henceforth.

### 4. Generation of Pseudo-real Data

0.4%–0.8% “activation” above the baseline was superimposed on resting state datasets described in the previous section. Area, location and time-course of “activation” were prescribed based upon those observed for real data. Pseudo-real data generated in this manner was used to compare different multi-run ICA methods for analysis of the data with noise structure, activation localization and activation magnitude similar to the real fMRI data.

Pseudo-real data (generated as described above) is referred to as D_preal_ later in the text.

### 5. Data Analysis

All the analysis steps were performed using custom MATLAB programs unless otherwise noted. Only the most superficial slice was used for the analysis since it contained the activity of interest, given the prior knowledge about anatomical locations of the representations of D1 and D3 in the primary somatosensory cortex.

#### 5.1. Preprocessing

Motion correction and coregistration between the individual runs within a session were performed using AFNI [23]. All the images were smoothed using a 3×3 Gaussian kernel with σ = 2 pixels. The time-courses were detrended by fitting and removing 3^rd^ order polynomials. Motion parameters were regressed out of the individual time-courses. In order to minimize the contribution due to non-neural sources, the contribution of the signals from the voxels belonging to skin was regressed out. To achieve this, regions of interest (ROIs) were manually drawn to obtain a skin mask. Temporal principal component analysis was performed on the signals from skin, and the principal components corresponding to the 5 largest eigenvalues were regressed out of all the time-courses. The resultant time-courses were low-pass filtered (0–0.1 Hz).

#### 5.2. Hypothesis-driven activation mapping

All the preprocessed runs within a session were averaged together, yielding an averaged dataset. Cross-correlation was obtained between the individual time-courses of the averaged dataset and a boxcar reference corresponding to the stimulus, to obtain a cross-correlation map for each session.

#### 5.3. Optimization of model order selection criterion

We assessed the performance of five model order estimation criteria (MDL, AIC, BIC, LAP and BSA) on simulated datasets (D_sim_). MELODIC was used for nIC estimation using MDL, AIC, BIC and LAP [15]. BSA (based on [13]) was implemented in MATLAB. The following steps describe the BSA method as used in this article:

1. A set of principal components P_0_ was obtained by performing PCA on all the images in a run.
2. For the k^th^ iteration, a set of principal components (P_k_) was obtained from bootstrapped samples from the image series. Each bootstrapped dataset consisted of ∼1/3 of the total images in the series.
3. Correspondence between members of P_0_ and P_1_ …. P_N_ (N =  number of bootstraps) was established. Hierarchical clustering (with the number of clusters forced to match the number of principal components) was used to establish correspondence between principal components.
4. Bootstrap stability of the components in P_0_ was estimated. We used1-|correlation-coefficient| as a measure of dissimilarity between the components obtained from the original dataset and bootstrap replicates.
5. A Gaussian-noise dataset was generated with the same dimension as that of the dataset on which nIC estimation was being performed. When temporal filtering/spatial blurring was applied to the data, the same filtering/blurring was applied to the noise dataset. Steps 1 through 4 were repeated for the noise dataset to estimate the stability of the principal components under the null hypothesis condition (i.e. when no structured variance is present in the data).
6. A Mann-Whitney U-test is used to estimate the number of stable components (p<0.05). The distributions compared using the Mann-Whitney U-test were 1) bootstrap stability of a given component, and 2) bootstrap stability of the *First* principal component of the Gaussian noise dataset.

To save computational time, only the first 100 principal components were used when establishing component correspondence (step 3). The actual number of stable components (as obtained by this method) was much less than 100, as described later in the text. The number of bootstraps was chosen to be 500 for the noise dataset and 100 for the dataset on which nIC estimation was being performed.

#### 5.4. ICA on real datasets

We used two approaches (ICA_avg_ and ICA_cat_) to analyze D_real_ datasets. As shown in the results section, BSA yielded optimal results on the synthetic data, and was used for nIC estimation on real data. For comparison, we also estimated nIC for real data using two popular ICA packages for fMRI (MELODIC [15] and GIFT) using their default nIC estimation methods (LAP and MDL). For ICA_cat_, we first used BSA to estimate the optimum nIC for each run of a particular session. We then set the model order equal to the largest nIC identified in a single run in a particular session. Group ICA for fMRI Toolbox (GIFT) was used to perform group ICA on all the runs for a given session (GIFT uses ICA_cat_ for performing group ICA when multiple datasets are supplied) [3]. To identify the task-related independent components, we obtained part correlation between the run-specific time-courses of individual independent components within a session, and the reference time-course. The independent component corresponding to maximum average part correlation with the reference time-course was chosen as the task-related component.

For ICA_avg_, we averaged all the preprocessed runs within a session using MATLAB and saved the averaged dataset as a NIFTI file (for compatibility with GIFT). nIC for the averaged dataset was estimated using BSA. GIFT was used to perform single-run analysis on the averaged dataset [3]. We used this approach to assess whether the improvement in CNR associated with averaging away non-phase coherent signal variations across the sessions could improve our ability to faithfully detect the activation patterns present in the data. The task-related component was identified as the component for which the associated time-course exhibited highest part correlation with the reference time-course.

Similarity between the ICA results (stimulus related ICA maps and associated time-courses) and the hypothesis-driven results (activation map and stimulus time-course) was assessed using correlation coefficients between the corresponding time-courses and maps, and Bland-Altman analysis [24].

#### 5.5. Comparison of multi-run ICA methods

D_preal_ datasets were used for comparison of ICA_avg_ and ICA_cat_. Since the true activation maps for the D_preal_ datasets were known, we were able to obtain true ROC curves corresponding to the two methods. These curves were used to compare the methods for multi-run analysis.

#### 5.6. Thresholding and display of maps

Activation maps (derived using ICA/cross-correlation analysis) were rescaled to spatial z-scores (number of standard deviations from the mean map). The resultant maps were thresholded (e.g. 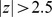), color coded and overlaid on anatomical images [25], [26]. These z-values do not reflect significance, and are used for visualization only [25], [26]. In cases where the area of activation is much smaller than the total brain area covered in the field of view, higher z-values of activated areas would indicate higher “contrast to noise” ratio of the activation map.

## Results

### 1. Optimization of model-order selection criterion

**Figure 1:**
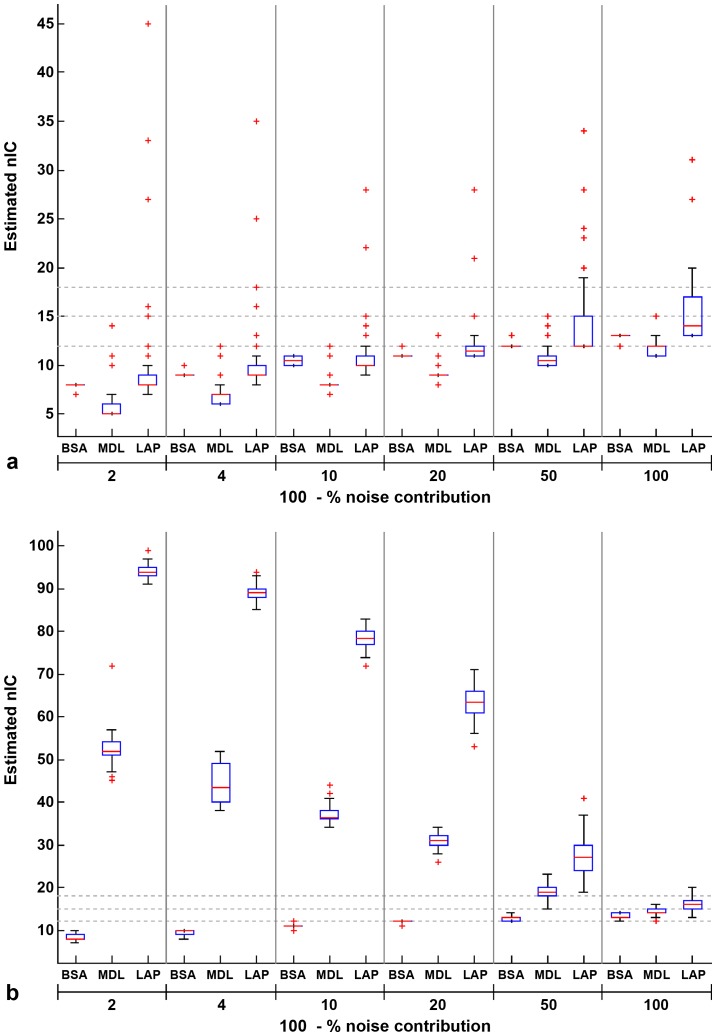
Comparison of nIC estimates for simulated data (with true nIC  = 15) using different approaches. Box and whisker plots for different noise levels and nIC estimation methods are shown. Red line in the middle of a box represents median nIC, whereas the boundaries of a blue box are the 25^th^ and 75^th^ percentile. The whiskers extend to the most extreme values that are not outliers, and the outliers are shown using red + signs. Here, outliers are defined as values that are above the 75^th^ percentile or below the 25^th^ percentile by a minimum difference of 1.5x(75^th^ percentile –25^th^ percentile). Bottom, middle and top grey dotted lines corresponds to nIC  = 12, 15 (true nIC) and 18. *a) nIC estimates without temporal filtering* –BSA results in the most stable estimates of nIC (smallest spread and number of outliers) for all noise levels, compared with the other methods. On average, nIC estimated using LAP method is closer to the true nIC (compared with BSA), but exhibits very high spread and number of outliers. *b) nIC estimates with temporal filtering (f<0.1* *Hz*): BSA results in the most stable and accurate estimates of nIC, compared with the other methods. Other methods result in highly exaggerated estimates of nIC, especially for high noise levels. Overestimation of nIC is most severe when the LAP method is used. These results indicate that BSA is compatible with temporal filtering which might be required to increase % variance contribution due to the sources of interest.

Figure 1 summarizes nIC estimates obtained using different strategies for different contributions due to Gaussian noise using box-and-whisker plots. AIC, MDL and BIC estimates were identical (which may be expected given their very similar mathematical forms), and therefore, we have only included MDL estimates in Figure 1.

Figure 1a shows the results in the situation where temporal filtering is not used to increase the contrast-to-noise ratio. BSA yields the most consistent estimates of nIC of all the methods across all noise levels: the maximum difference between 25^th^ and 75^th^ percentile values is 1, and the detected outliers have a maximum difference of 1 from the median value for BSA. LAP estimates have the highest inconsistency, as apparent from larger spread and extreme values of the outliers. nIC estimated using LAP shows the least bias (i.e., the average difference between LAP estimate and true nIC is the lowest, compared with other methods). However, LAP-derived nIC estimates also show the greatest amount of variability, and hence least reliability. The performance of the other methods (BIC, AIC and MDL) is inferior to the performance of BSA method in terms of bias and consistency. In general, low CNR results in underestimation of nIC (Figure 1a)

Figure 1b illustrates the dangers of nIC estimation in the presence of temporal filtering, particularly in the setting of low SNR. When temporal filtering is performed, LAP, AIC, MDL and BIC yield highly exaggerated estimates of nIC when noise levels are high (Figure 1b, AIC and BIC based estimates were equal to MDL based estimates in all cases). In contrast, BSA is remarkably resistant to inflation of the estimated nIC in temporally filtered data. In fact, temporal filtering results in a slight reduction in bias of BSA estimates for all noise levels (∼0.5, on average).

These results suggest that BSA provides the most consistent and stable estimates for nIC and does so regardless of whether temporal filtering is performed. These estimates can be further improved by incorporating prior information about the frequency range of the signals. BSA was used for obtaining the results described in the rest of the article, unless otherwise noted.

### 2. Hypothesis-driven activation mapping

**Figure 2:**
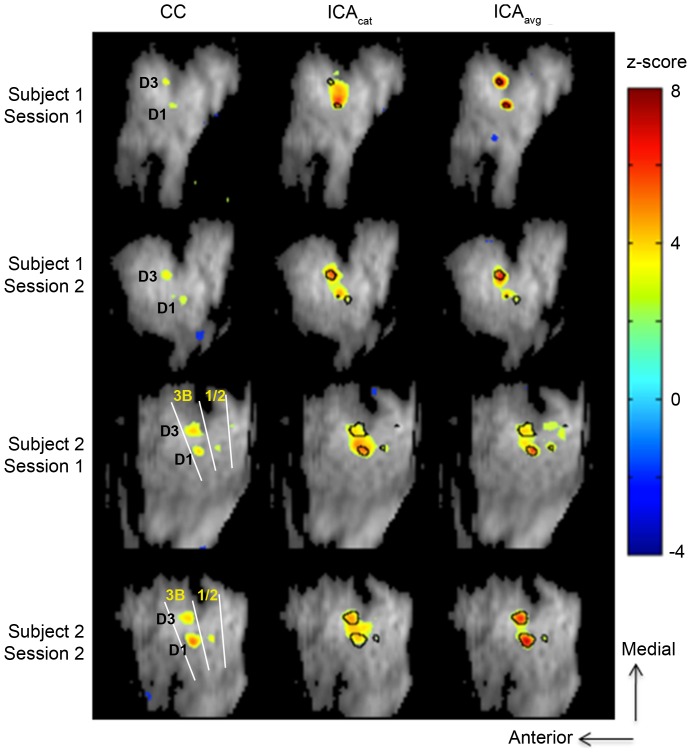
Activation maps obtained using cross-correlation analysis (CC), ICA_cat_ and ICA_avg_ (|z|>2.5). In general, ICA provides greater contrast (in terms of z-score) between activated and non-activated brain regions. Compared with ICA_cat_, ICA_avg_ provides greater functional specificity and contrast. The results are consistent across subjects and sessions. These maps correspond to nIC estimated using the bootstrap stability analysis approach (nIC  =  (9.8±1.2)).

Tactile stimulation resulted in activation of expected regions in somatosensory cortex for all monkeys and sessions. For additional information about expected activation, see [21]. As seen in Figure 2 (column 1), hypothesis driven analysis (based on voxel-wise correlation with the stimulus time-course) detects activation in area 3b of somatosensory cortex corresponding to digits 1 and 3, as expected. Some activation in area 1 is also observed. Average percentage signal decrease corresponding to the voxel exhibiting highest correlation with the stimulus time-course ranged between 0.35% and 0.8%. The difference map (obtained after subtracting average signal intensity during stimulation from average signal intensity during rest) explained 1.5–3.5% of the total variance in the smoothed and detrended data. However after including the remaining preprocessing steps (regressing out motion parameters, principal components from skin and filtering), the variance explained by the difference maps ranged between 2.5 and 9.2% due to removal of some of the task-unrelated variance.

### 3. ICA on the real data

Unless otherwise noted, the model order selection was made based upon BSA. Application of BSA to the preprocessed data yielded a stable estimate of the number of components across all runs and sessions (9.8±1.2 for ICA_cat_, 9.75±1.25 for ICA_avg_, pooled over all subjects and sessions. Consistent with the simulation results, the model order estimated using LAP (default method used in MELODIC [15]) and MDL (default method used in GIFT) yielded exaggerated estimates of nIC (ranging between ∼50 and ∼170; for one subject, local minima of the MDL function were found for nIC between 11 and 15. However, the global minima in those cases always occurred for nIC >160). ICA performed with this model order resulted in overfitting, and the individual components were found to be very sparse, with highly localized foci of “activation”. Results of ICA_avg_ with nIC  = 176 (as suggested by MDL) on one of the datasets are shown in Figure S1.

Stimulus-related independent components obtained for ICA_cat_ and ICA_avg_ are shown in Figure 2, columns 2 and 3 respectively. Visual inspection suggests that the ICA_cat_ and ICA_avg_ generate spatial modes consistent with classically derived activation maps. In general, ICA maps had greater functional contrast (expressed by the z-score in the activated regions), compared with correlation-based activation maps. ICA_avg_ yields the greater functional contrast of the two ICA approaches. Two factors may contribute to the improved CNR in the ICA maps: the noise reduction associated with PCA filtering in first step of ICA, and ICA's ability - in contrast to cross-correlation based analyses - to separate the sources. The stimulus-related independent components obtained from ICA_avg_ exhibit greater similarity (measured in terms of cross-correlation) with the hypothesis-driven activation map, in comparison with ICA_cat_ (0.63±0.16 vs 0.50±0.20). Additionally, the temporal profiles associated with the stimulus-related independent component obtained using ICA_avg_ showed greater correlation with the stimulus time-course, compared with that corresponding to ICA_cat_ (0.61±0.07 vs 0.51±0.17). These findings suggest that greater functional contrast and spatial/temporal accuracy can be achieved when ICA_avg_ is used (for the case of phase-synchronized stimulus-driven response across the datasets to be averaged), where spatial/temporal accuracy is estimated by similarity of the maps/time-courses with those associated with hypothesis-driven analysis.

**Figure 3:**
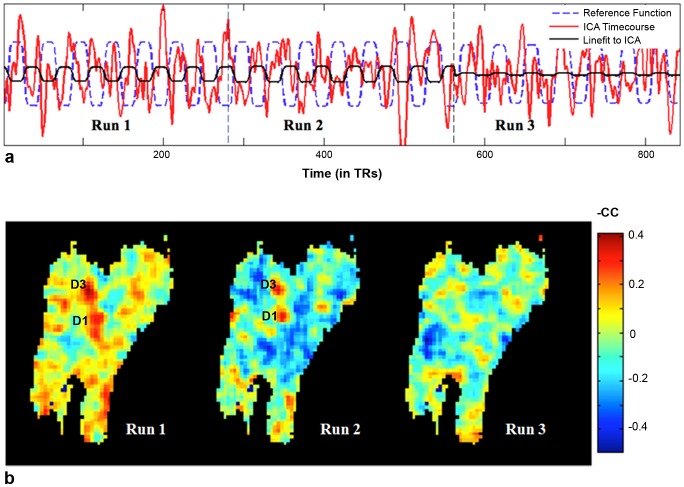
Run-specific changes in activation as captured by ICA and hypothesis-driven analysis. a) Run-specific time-courses for the stimulus-related independent component obtained using ICA_cat_ capture differences in stimulus-related contribution. “linefit to ICA” represents least squares approximation of the ICA time-course in terms of the reference function. b) These variations are in agreement with the maps obtained using hypothesis-driven (correlation-based) analysis. The images are shown with reversed contrast, so that activated pixels appear hot.

**Figure 4:**
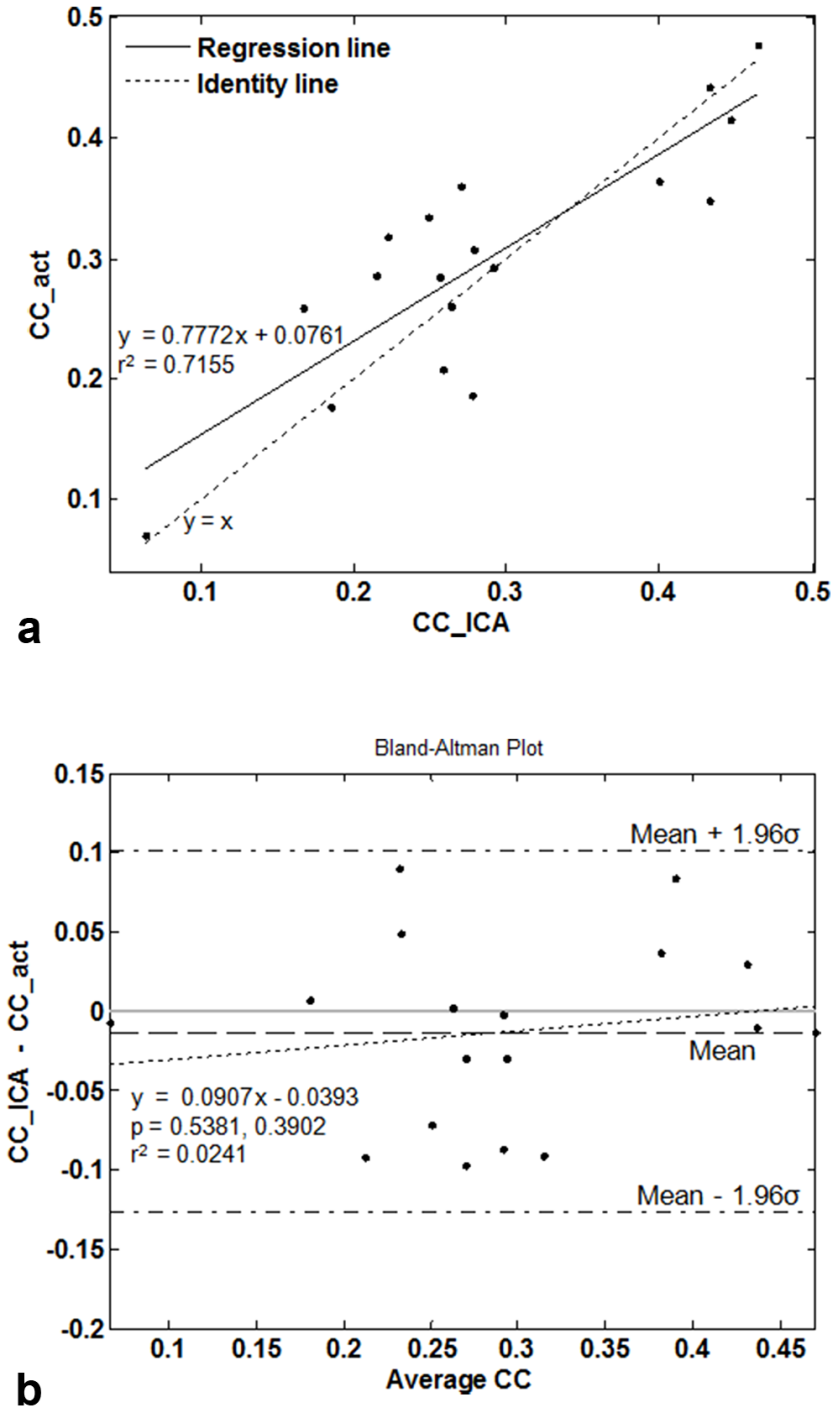
Strong agreement between model-free data-driven ICA and cross-correlation with stimulus model. a) A linear relationship is observed between 1) similarity (as measured by correlation) of the temporal profile of the stimulus-related IC and stimulus function (CC_ICA), and 2) average correlation of activated voxels (based on correlation-based activation maps) with stimulus function (CC_act). b) Bland–Altman plots and fitted linear trends of mean vs. difference between CC_ICA and CC_act. The difference shows negligible bias and an insignificant linear trend, suggesting strong agreement between CC_ICA and CC_act.

Figure 3 shows run-specific time-courses associated with the stimulus-related independent component obtained using ICA_cat_ (3 runs for one session, separated by vertical lines), along with the stimulus-related component of the time-courses (obtained using linear fitting). As can be seen, run-specific variations in stimulus-dependent response can be captured using ICA_cat_. Run-specific correlation-based activation maps for these runs are displayed in Figure 3 (row 2). Visual comparison between rows 1 and 2 of Figure 3 suggests *improved* functional contrast (for hypothesis-driven analysis) associated with greater stimulus-dependent temporal contribution of the stimulus-related independent component. Both correlation (Figure 4a) and Bland-Altman analyses (Figure 4b) confirm the strong agreement between the ICA and hypothesis driven measures, i.e. 1) similarity (as measured by correlation) of the temporal profile of the stimulus-related IC and stimulus function, and 2) average correlation of activated voxels (based on correlation-based activation maps) with stimulus function. Figure 3 and 4 suggest that differences in run-specific time-courses of the stimulus-related independent component faithfully capture variation in stimulus-driven response across different runs.

**Figure 5:**
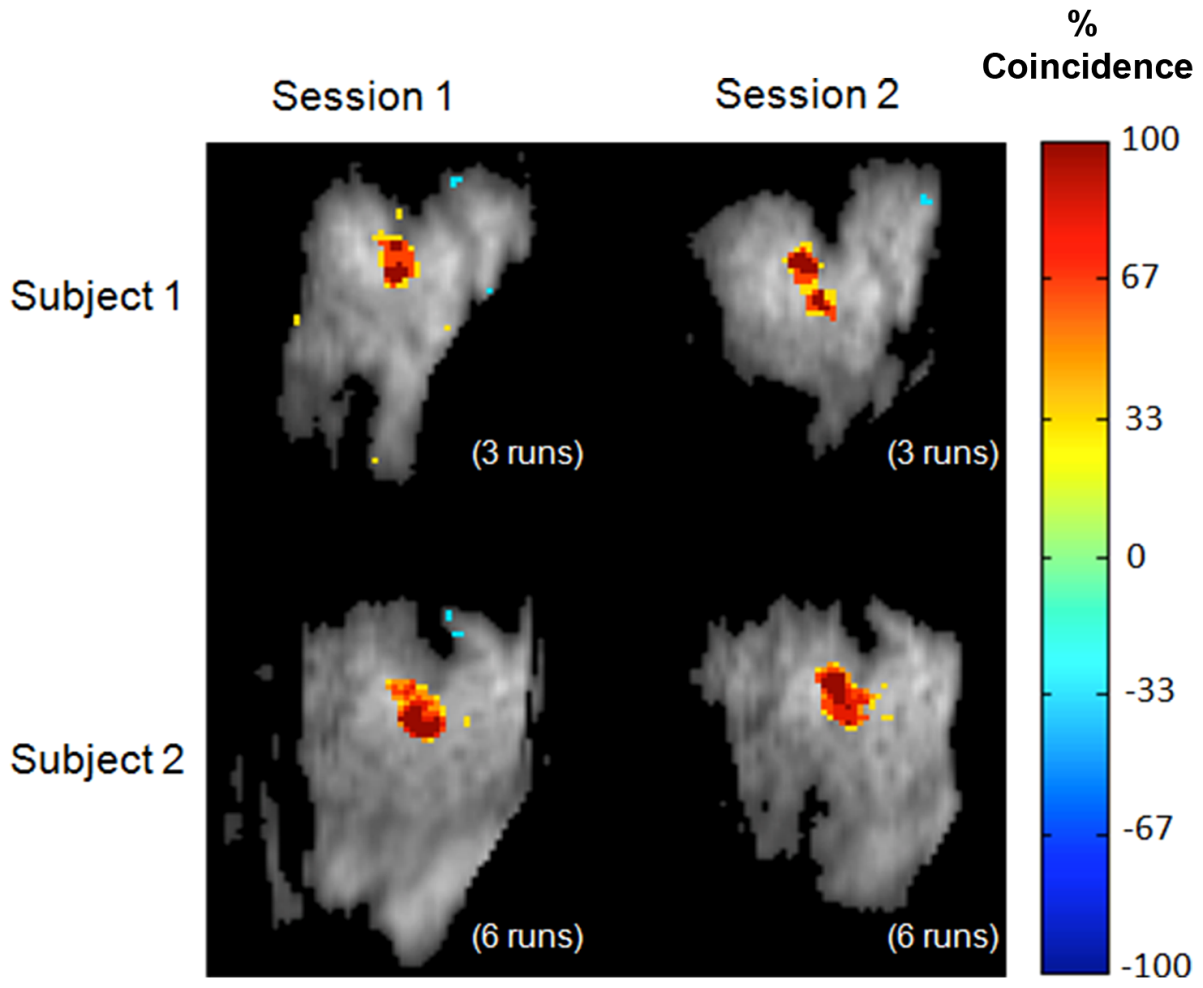
High run-to-run reproducibility of activation maps obtained using ICA. Coincidence maps, representing the percentage of single runs within a session for which ICA identified a given voxel as activated, are shown in the figure. To identify activated voxels, run-specific maps were converted to z-scores and thresholded (|z|>2.5). Negative values indicate that the voxel appears with negative intensity.

Coincidence maps derived from the stimulus-related maps for individual runs (obtained using ICA_cat_) were found to be highly reproducible within and between the sessions and animals (Figure 5).

### 4. Comparison of multi-run ICA strategies using pseudo-real data

**Figure 6:**
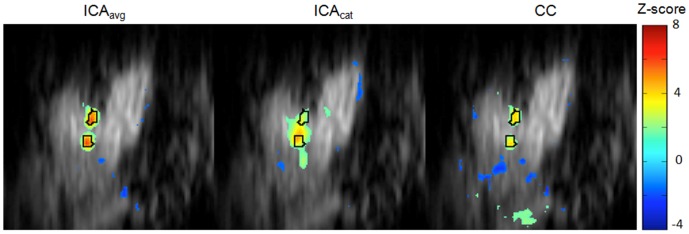
Stimulus-related IC maps obtained using different multi-run ICA approaches, compared with hypothesis-driven activation map. Consistent with the results for real datasets ICA_avg_ provides more specific maps and higher functional contrast, compared with ICA_cat_ and cross-correlation-based hypothesis-driven analysis. A lower z-threshold (|z|>1.5) was chosen to highlight the differences in specificities of these approaches.

**Figure 7:**
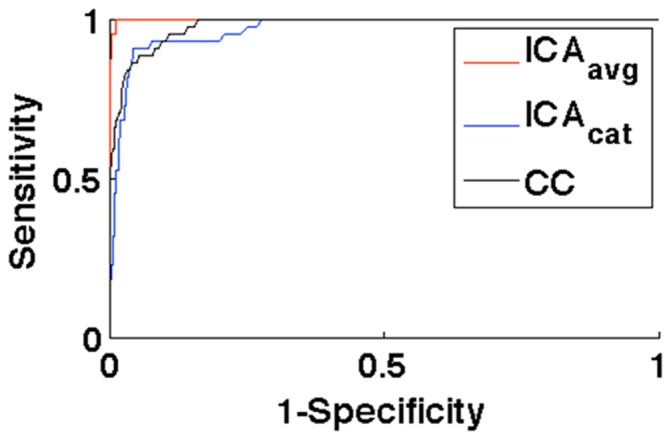
ROC curves (pseudo-real data with 0.4% activation above the baseline. ICA_avg_ provides more sensitivity for a given level of specificity, compared with ICA_cat_ and cross-correlation based hypothesis-driven analysis. This observation is consistent with Figure 6 as well as the results obtained for real datasets (Figure 2).

Figure 6 shows task-related IC maps obtained from pseudo-real datasets with 0.4% activation using three different multi-run ICA approaches. True activation boundaries for D1 and D3 are encircled in black. Consistent with the maps obtained from real data, the visual comparison reveals that the spatial specificity of the task-related map obtained using ICA_avg_ is superior to that using ICA_cat_ and generates fewer spurious areas compared with the cross-correlation based hypothesis-driven analysis. Quantitative comparison of the detection power of these approaches using ROC analysis (Figure 7) confirms that ICA_avg_ provides more sensitivity for a given level of specificity, compared with ICA_cat_. Furthermore, the area under the ROC curve for ICA_avg_ is also greater than that for hypothesis-driven, correlation-based activation mapping.

## Discussion

The research presented in this article has three main contributions. First, we address the issue of selection of optimal model order for ICA of fMRI. We compared five approaches for data-driven model order estimation (four of which have been implemented in widely available ICA software) and conclude that model order selection based upon bootstrap stability of principal components significantly outperforms more commonly used methods (AIC, MDL, BIC and LAP) for a wide range of CNRs. Secondly, we push the limits of sensitivity of ICA by demonstrating that it can be used to reproducibly detect weak (as small as 0.4% above the baseline) and focal neural responses in fMRI that are produced in response to tactile stimulation of individual digits under anesthesia in a completely data-driven way. Third, we compare two different approaches for group/multi-run ICA.

There has been no consensus on model order selection criteria for ICA and it still remains an area of active research. Several approaches have been proposed to select the model order. Some studies have chosen the model order based upon the fraction of total variance explained by the first few principal components of the data (e.g. more than 99.7%). Other studies have utilized a model order based upon arbitrary selection, e.g. [11]. Such criteria have no relationship with the inherent dimensionality of the data. Information theoretic criteria such as AIC and MDL have also been used to estimate the number of independent sources. These methods are more objective, but have been reported to overestimate the number of components [10]. Model order is a very important parameter for “blind” source separation, as it can heavily influence the findings. Thus, inclusion of too many principal components in the data-reduction step results in sparse components, with smaller regions of activation [9], [27]. Also, the resultant components have been reported to be less reliable [27]. In contrast, smaller model orders tend to result in less focal component maps and may not reflect the true underlying complexity of the source signals. While manual adjustment of nIC, possibly hypothesis/model driven, may allow one to arrive at the number of components that yields “desired” results (i.e. similar to an expected activation pattern), this defeats the very purpose of data driven analysis and biases the results in favor of the expected/desired result. Our simulations suggest that BSA outperforms commonly used nIC estimation criteria in terms of accuracy and reliability. Also, and in contrast to the methods most commonly used for nIC estimation, we found that BSA performance is not degraded when temporal filtering is performed to increase the CNR based upon the prior knowledge of the frequency bands of importance (Figure 1b). BSA is resistant to this effect because it processes the real data and the data under the null hypothesis condition using the same steps. Accuracy, reliability and the fact that this approach is relatively insensitive to the preprocessing steps make it a desirable method for model order selection.

**Figure 8:**
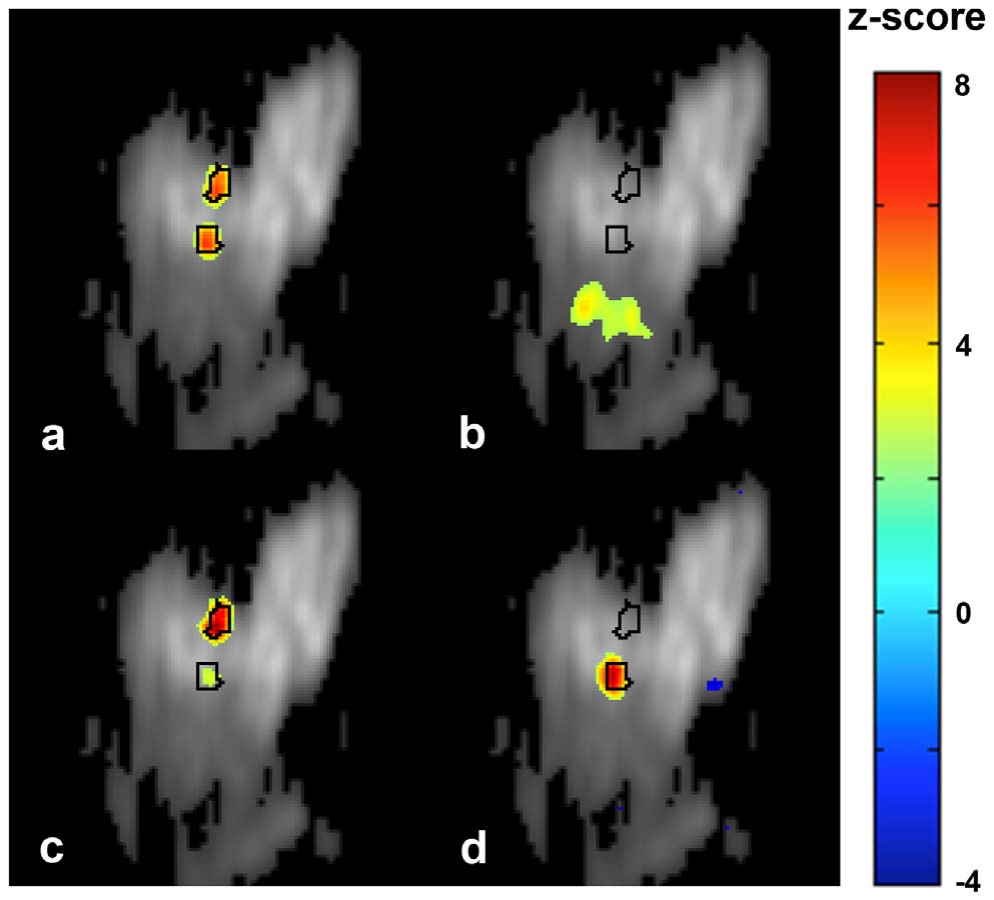
Task-related components obtained from pseudo-real data using different model orders. a) Activation-related map obtained with nIC  = 10 (as suggested by BSA) corresponds well with the ground truth. b) Spatial map associated with time-course exhibiting highest part correlation with stimulus time-course when setting nIC  = 5 (i.e. under-estimate of nIC). Underestimation of nIC leads to loss of a spatial component corresponding to the “ground truth” activation. c, d): Over-estimation of nIC (nIC  = 50) splits the ground truth activation map into 2 components.

To demonstrate the effects of over/under estimation of nIC on the estimated sources, we performed ICA_avg_ on pseudo-real data (setting activation at 0.4% above the baseline) with different model orders. For nIC  = 10 (as suggested by BSA), the activation-related component shows excellent agreement with the “ground truth” (outlined regions, Figure 8, a). However, for nIC  = 5, none of the resultant components corresponds to the “ground truth” activation map. Indeed the component whose time-course exhibits the highest part-correlation with the stimulus time-course shows no spatial correspondence with the “ground truth” map (Figure 8, b). This is expected because underestimation of nIC results in loss of components that contribute relatively smaller variance to the data, and the simulated activation is localized to a small area and is weak (0.4% above the baseline). In fact, the “ground truth” does not appear in one of the ICA maps for any model order smaller than 9. Figures 8 c and d demonstrate effect of overestimation of nIC on ICA results. Two components with high temporal correlation with stimulus time-course (part correlation >0.3) are observed when nIC  = 50, suggesting that the task-related component is split into two sub-components. This result is in agreement with previous work that suggest that increasing nIC results in sparser components [27]. This subdivision of “activated area” does not correspond to greater functional specificity of the maps and represents purely the effect of overfitting, because the “stimulus-related” activity was added with equal magnitude and phase to all the voxels within the outlined regions in Figure 8.

The degradation of performance of conventional approaches to nIC estimation (AIC, MDL, BIC and LAP) when using pre-filtered data is very dramatic and indicates that their use on preprocessed data requires great caution. This degradation of performance in the presence of temporal filtering may be attributed to violation of the underlying assumptions of common implementations of those approaches. For example, the commonly used implementation of AIC and MDL, as proposed in [16] assumes independent and identically distributed (i.i.d.) samples, which is violated due to spatial blurring and the intrinsic point-spread function of fMRI. [28] proposed a procedure for obtaining effectively i.i.d. samples by downsampling the data, resulting in improved estimation of nIC (MDL, as implemented in GIFT, utilizes this approach). Another assumption in the formulation of AIC and MDL proposed in [16] is that the additive unstructured noise is white. Temporal filtering adds structure to the noise. Therefore, the correlation matrix of the data may not be partitioned into white and colored components, as assumed in [16]. This issue is not encountered in the case of BSA, since the only assumption involved is that the principal components corresponding to the underlying sources are stable, and the randomized data (used to generate null-distribution) can be filtered equivalently in cases where filtering is performed on the real data.

The two group/multi-run ICA methodologies (ICA_avg_ and ICA_cat_) have their strengths and drawbacks, and which approach is optimal will depend on the contexts/constraints: In situations where inter-run/session/group differences are unimportant, neuronal activity of interest is coherent across the runs/session/groups, and spatial localization of the neuronal activity of interest is the main objective, ICA_avg_ offers better performance with less computational load. The results we obtained using pseudo-real data suggest that of the three approaches compared, ICA_avg_ has the greatest area under the ROC curve. One possible explanation for this could be the higher effective CNR achieved due to averaging. Averaging not only reduces the contribution due to thermal noise, but also other components of noise such as respiratory and cardiac sources of variance, making stimulus-driven activity more likely to be fully captured by the first few principal components. Indeed ROC analysis indicates that ICA_avg_ performance is superior to hypothesis-driven activation mapping (cross correlation based activation mapping). This may be explained by the fact that ICA inherently favors separation of sources of structured variance if they are non-Gaussian and independent.

Despite its high sensitivity for a given level of specificity for the experiments conducted in a block-design setting, ICA_avg_ cannot provide any information about run-specific differences in IC maps or activation time-courses. Also, the ICA_avg_ approach is incompatible with experimental paradigms where brain activity changes of interest are not synchronized/phase-locked across the runs/sessions/subjects (e.g. resting state or random event-related fMRI experiments). A concatenation-based multi-run/group ICA approach needs to be used in such circumstances. ICA_cat_ has been popular for concatenation-based ICA analysis due to its lower computational burden and ability to identify run/subject specific differences in activity. ROC analysis suggests that ICA_avg_ provides superior performance to ICA_cat_. Consistent with the ROC results, visual comparison suggests that the task-related IC map obtained using ICA_cat_ is more “blurred” compared with that obtained using ICA_avg_, possibly as a result of an underestimation of nIC. This underestimation of nIC may arise from the presence of task-independent but run-specific ICs reflecting sources of variance that may not contribute evenly across the runs. In other words, run-specific underlying independent components may be present. Estimation of nIC on a run-by-run basis and taking their maximum would not account for unique stable modes for different runs, and might therefore lead to underestimation of nIC. This problem might be overcome by performing nIC estimation on the concatenation of the individual runs within a session. We estimated nIC on the concatenation of all the runs for the simulated datasets and found that the nIC estimates for the concatenated dataset (∼19) was higher than that estimated for any of the runs individually (∼11), confirming the explanation provided above. The only potential drawback of this approach is higher computational burden and a consequent increase in processing time (∼6-fold in this case).

We have used several preprocessing steps for noise removal in order to increase effective CNR. This serves different purposes: First, it increases the fraction of stimulus-related variance in the data, making it more likely to be captured by the first few principal components, and less likely that those principal components capturing stimulus-related activity are lost in one of the reduction steps. Secondly, an increase in CNR translates into better spatial and temporal accuracy of the independent component maps. These preprocessing steps are not a standard part of many ICA analyses. However, a few animal studies have utilized filtering as a preprocessing step, e.g. [7]. A lower frequency cutoff for the low-pass filter could have been chosen, since the basic frequency of the stimulus function was 0.0167 Hz. We chose to use 0.1 Hz because of its relevance for resting state fMRI studies.

Most functional connectivity studies have highlighted the presence of large-scale networks (for example default mode network) [29], [30]. However, it is well understood that much smaller functional units exist in the brain such as cortical columns, that may be detected using fMRI [22], [31]. This leads to the question of whether one can identify resting state connectivity networks at that level of functional specificity. Our study demonstrates that very localized and weak neuronal responses can be detected in a completely data-driven way using fMRI, making it reasonable to speculate that it may be possible to detect highly localized networks with coherent spontaneous activity. Further multi-modal investigations along these lines will play a crucial role in 1) determining whether such local networks are present, 2) assessing whether fMRI is sensitive enough to detect those networks and 3) establishing the neurophysiological importance of such networks.

In conclusion, this study addresses one of the most fundamental issues involved in data-driven ICA, i.e. nIC estimation, and suggests an approach that outperforms commonly used methods. Additionally, it pushes the limit of sensitivity of ICA of fMRI, and adds to the applications for which it may be used.

## Supporting Information

Figure S1 **Effect of overestimation of nIC.** Choosing nIC  = 176, as suggested by MDL, results in sparse components with localized “hot spots”, illustrating the effect of overestimation of nIC.
(TIF)

Checklist S1 **ARRIVE Checklist.**
(DOC)
