# Supplementary figures and images for: Robust Data Driven Model Order Estimation for Independent Component Analysis of fMRI Data with Low Contrast to Noise
Source: PLoS One. 2014 Apr 30;9(4):e94943. doi: 10.1371/journal.pone.0094943 (PMC4005775; doi:10.1371/journal.pone.0094943)

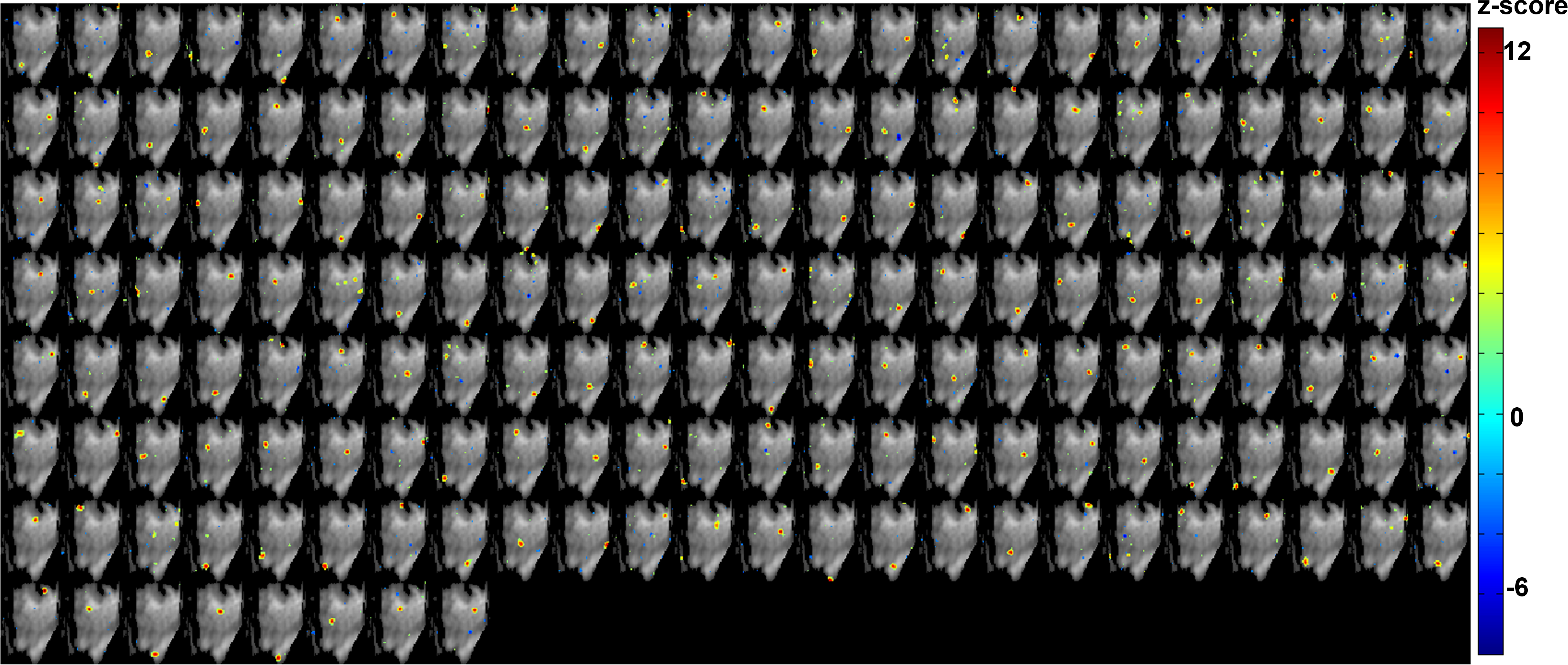

Supplement: Figure S1 — Effect of overestimation of nIC. Choosing nIC = 176, as suggested by MDL, results in sparse components with localized “hot spots”, illustrating the effect of overestimation of nIC. (TIF) [file pone.0094943.s001.tif]
